# A First Report of Molecular Typing, Virulence Traits, and Phenotypic and Genotypic Resistance Patterns of Newly Emerging XDR and MDR *Aeromonas veronii* in *Mugil seheli*

**DOI:** 10.3390/pathogens11111262

**Published:** 2022-10-29

**Authors:** Abdelazeem M. Algammal, Reham A. Ibrahim, Khyreyah J. Alfifi, Hanaa Ghabban, Saad Alghamdi, Ahmed Kabrah, Ahmed R. Khafagy, Gehan M. Abou-Elela, Nermeen M. Abu-Elala, Matthew Gavino Donadu, Reham M. El-Tarabili

**Affiliations:** 1 Department of Bacteriology, Immunology, and Mycology, Faculty of Veterinary Medicine, Suez Canal University, Ismailia 41522, Egypt; 2 National Institute of Oceanography and Fisheries, Cairo 11516, Egypt; 3 Department of Biology, Faculty of Science, University of Tabuk, Tabuk 71421, Saudi Arabia; 4 Laboratory Medicine Department, Faculty of Applied Medical Sciences, Umm Al-Qura University, Makkah 21955, Saudi Arabia; 5 Department of Aquatic Animal Medicine and Management, Faculty of Veterinary Medicine, Cairo University, Giza 12211, Egypt; 6 Faculty of Veterinary Medicine, King Salman International University, El Tor 46612, Egypt; 7 Department of Biomedical Sciences, University of Sassari, 07100 Sassari, Italy; 8 Department of Medical, Surgical and Experimental Sciences, University of Sassari, 07100 Sassari, Italy

**Keywords:** *A. veronii*, sequence analysis, virulence, XDR, pathogenicity, antimicrobial resistance genes

## Abstract

*Aeromonas veronii* is associated with substantial economic losses in the fish industry and with food-borne illness in humans. This study aimed to determine the prevalence, antibiogram profiles, sequence analysis, virulence and antimicrobial resistance genes, and pathogenicity of *A. veronii* recovered from *Mugil seheli*. A total of 80 fish were randomly gathered from various private farms in Suez Province, Egypt. Subsequently, samples were subjected to clinical, post-mortem, and bacteriological examinations. The retrieved isolates were tested for sequence analysis, antibiogram profile, pathogenicity, and PCR detection of virulence and resistance genes. The prevalence of *A. veronii* in the examined *M. seheli* was 22.5 % (18/80). The phylogenetic analyses revealed that the tested *A. veronii* strains shared high genetic similarity with other *A. veronii* strains from India, UK, and China. Using PCR it was revealed that the retrieved *A. veronii* isolates harbored the *aer*A, *alt*, *ser*, *omp*AII, *act*, *ahp*, and *nuc* virulence genes with prevalence of 100%, 82.9%, 61.7%, 55.3%, 44.7%, 36.17%, and 29.8%, respectively. Our findings revealed that 29.8% (14/47) of the retrieved *A. veronii* strains were XDR to nine antimicrobial classes and carried *bla*_TEM_, *bla*_CTX-M_, *bla*_SHV,_ *tet*A, *aad*A1, and *sul*1 resistance genes. Likewise, 19.1% (9/47) of the obtained *A. veronii* strains were MDR to eight classes and possessed *bla*_TEM_, *bla*_CTX-M_, *bla*_SHV,_ *tet*A, *aad*A1, and *sul*1 genes. The pathogenicity testing indicated that the mortality rates positively correlated with the prevalence of virulence-determinant genes. To our knowledge, this is the first report to reveal the occurrence of XDR and MDR *A. veronii* in *M. seheli*, an emergence that represents a risk to public health. Emerging XDR and MDR *A. veronii* in *M. seheli* frequently harbored *aer*A, *alt*, *ser*, *omp*AII, and *act* virulence genes, and *bla*_TEM_, *sul*1, *tet*A, *bla*_CTX-M_, *bla*_SHV_, and *aad*A1 resistance genes.

## 1. Introduction

The fast-growing demand for seafood presents a significant challenge for the enhancement of fisheries and aquaculture production worldwide. In 2014, the contribution of aquaculture to the human food supply overtook the production of natural water resources for the first time [1]. Egypt is the leading African country in terms of aquaculture production, with about 1.8 million tons of aquatic animal production, including freshwater and marine fish, shellfish, and crustaceans [2]. Mullets belong to the *Mugilidae* family, consisting of more than 72 species from 17 fish genera; *Mugil cephalus* (gray mullet) and *Mugil seheli* (bluespot mullet) are frequently cultured in the Suez Canal region. Mullet species occupy third place in terms of fish production in Egypt [3,4]. *M. seheli* is a commercially important fish species in the Suez Bay and in Egypt, although it grows slowly. It has a larger market price in Egypt than other mullets, due to its highly regarded flavor [5].

Infectious bacterial diseases adversely affect the aquaculture industry through direct economic losses related to fish mortality, and indirectly due to costs associated with disease control and reduction of production [3,4,6]. *Aeromonas* infection was previously reported in Nile tilapia (*Oreochromis niloticus*) [6], Chinese longsnout catfish (*Leiocassis longirostris günther*) [7,8], freshwater goldfish (*Carassius auratus*) [9], and catfish (*Ictalurus punctatus*) [10], resulting in severe economic losses in the fish industry and threatening public health [11]. *A. veronii* is categorized into two subspecies, *veronii* and *sobria* [12]. *A. veronii* is frequently incriminated in marine fish hemorrhagic septicemia [10]. *A. veronii* infection causes food-borne illness in humans, characterized by diarrhea, gastroenteritis, and sepsis [11,13]. The morbidity rate of *A. veronii* infection in fish was observed to be higher in summer when the water temperature is over 18 °C, usually reaching its peak in July [12].

The identification of *Aeromonas* species by traditional techniques is difficult due to the lack of a specific biochemical scheme to differentiate between them. Hence, the use of molecular assays provides more reliable identification and limits the incongruities associated with the biochemical identification of these pathogens [8,13]. Polymerase chain reaction (PCR) is an essential laboratory test for accurate and prompt bacterial identification and the investigation of virulence genes. *16SrRNA* and the housekeeping genes are the most common target genes for identification of *Aeromonas* species [14]. *Aeromonads* have various virulence determinants that empower them to overcome host defense mechanisms. Various toxins and enzymes are included among these virulence factors, including aerolysin (aer), cytotonic enterotoxins (act and alt), serine proteases (ser and aph), nuclease (nuc), and outer membrane proteins (ompAI and ompAII). These virulence determinants are encoded by specific virulence genes that regulate the potential pathogenicity of *A. veronii* [15,16,17]. *A. veronii* infection has been linked to widespread fish mortality in Egypt [18].

Antimicrobial resistance has increased worldwide due to the widespread improper use of antibiotics. Moreover, antibiotic residues in different fish products are considered a public health threat [19,20,21,22]. Antibiotic residues in aquaculture products may harm human health by suppressing or eliminating beneficial bacterial flora in the gastrointestinal tract (GIT) [23,24,25]. *A. veronii* has public health and epidemiological importance as a primary pathogen of fish, due to the emergence of MDR strains.

Although several previous studies have clarified the incidence of *A. veronii* among different fish species, to the best of our knowledge, no previous reports have revealed the emergence of *A. veronii* in *M. seheli*. The current study aimed to investigate the prevalence, antibiogram profile, sequence analysis, virulence and antimicrobial resistance genes, and pathogenicity of *A. veronii* recovered from *M. seheli*.

## 2. Materials and Methods

### 2.1. Animal Ethics

The handling of fish and all the experiments were approved by the Animal Ethics Review Committee of Suez Canal University (AERC-SCU), Egypt.

### 2.2. Sampling

A total of 80 moribund fish (*M. seheli* with an average body weight of 60 ± 5 g) were randomly collected from various private farms in Suez Governorate, Egypt, between July and September 2020. The collected fish were rapidly transported in aerated sealed plastic bags to the microbiology laboratory at the National Institute of Oceanography and Fisheries (NIOF), Suez, Egypt, for clinical and bacteriological examination.

### 2.3. Clinical and Postmortem Examinations

The naturally infected *M. seheli* were screened for detection of any abnormalities. The clinical inspection was carried out as previously described [26]. Necropsy was performed on moribund *M. seheli* [27].

### 2.4. Isolation and Identification of A. veronii

A loopful of the obtained samples (liver, kidney, and gills) was streaked directly onto tryptic soy agar (TSA) and *Aeromonas* isolation medium base (supplemented with ampicillin) (Oxoid, Hampshire, UK) and incubated at 28 °C for 18–24 h. [28,29]. The identification of *A. veronii* was performed using Gram’s staining, culture characteristics, hemolysis on blood agar, and biochemical characterization (oxidase, catalase, methyl red, Voges–Proskauer, citrate utilization, gelatin liquefaction, casein, starch liquefaction, sugar fermentation, H_2_S production, urea hydrolysis, bile esculin hydrolysis, and nitrate reduction tests). Additionally, the identification of *A. veronii* was confirmed by PCR detection of the *16srRNA* gene as previously stated [30], and gene sequencing was carried out.

### 2.5. 16S rRNA Gene Sequencing and Phylogenetic Analyses

PCR amplification of the *16S rRNA* gene was performed for all recovered *A. veronii* isolates. The retrieved *A. veronii* strains displayed congruence in their phenotypic features. Consequently, the PCR products of three isolates (chosen at random) were subjected to direct sequencing in both directions following purification using a PureLink PCR-Product purification kit (Thermo-Fisher Scientific, Bremen, Germany). The obtained sequences were placed in the GenBank with accession numbers MW831507, MW836109, and MW599727. Multiple alignments were performed on the obtained sequences. The phylogenetic tree was established according to the neighbor-joining approach with 1000 bootstrap resampling using MEGA X software [31,32].

### 2.6. Antibiogram of the Recovered A. veronii Isolates

The antimicrobial susceptibility of the obtained *A. veronii* isolates was examined using the disc diffusion method on Mueller–Hinton agar (Oxoid, UK). Thirteen antimicrobial agents were tested, including piperacillin/tazobactam (TZP, 100/10 μg), ampicillin (AMP, 10 μg), amoxicillin/clavulanic acid (AMC, 30 μg), ceftriaxone (CRO, 30 μg), cefotaxime (CXT, 30μg), sulfamethoxazole/trimethoprim (SXT, 25 μg), streptomycin (S, 10 μg), polymyxin B (PB, 300U), tetracycline (TE, 10 μg), chloramphenicol (C, 30 μg), rifamycin SV (RF, 30 μg), erythromycin (E, 15 μg), and ciprofloxacin (CIP 10 μg) (Oxoid, UK). The results were interpreted as previously described in CLSI guidelines [33]. All plates were incubated at 37 °C for 24 h. The *E. coli*-ATCC25922 strain was implemented as a reference. *A. veronii* isolates were classified as extensively drug-resistant (XDR: resistant to one or more antibiotics in all tested classes, except 1 or 2 classes) or multidrug-resistant (MDR: resistant to ≥ one agent in ≥ 3 antimicrobial classes), as previously described [34]. Furthermore, the multiple antibiotic-resistance index values (MAR index: number of antimicrobial agents to which the isolates are resistant/total number of tested antimicrobial agents) were estimated [35].

### 2.7. Determination of Virulence and Antimicrobial Resistance Genes in the Recovered A. veronii Strains

PCR was employed to determine the virulence-determinant genes *(aer*A, *ser*, *act*, *alt*, *ahp*, *nuc*, and *omp*AII*)* and antimicrobial-resistant genes (*bla*_TEM_, *bla*_SHV_, *bla*_CTX-M_, *aad*A1, *sul*1, and *tet*A) in the retrieved *A. veronii* strains. DNA extraction was carried out using the PureLink DNA extraction kit (Thermo-Fisher Scientific, Bremen, Germany/Cat. No. A29790). Negative controls (DNA-free) and positive control strains (provided by the AHRI, Dokki, Egypt) were included in the PCR assay. The obtained PCR products were separated on agar gel by electrophoresis. Afterwards, the gel was photographed. The primer sequences (Thermo-Fisher Scientific, Karlsruhe, Germany) and cycling conditions are presented in Table 1.

### 2.8. Pathogenicity Test

#### 2.8.1. Fish Sampling and Accommodation Period

Approximately 180 apparently healthy *Tilapia zillii* (weighting 45 ± 10 g) were collected from private farms in Suez Governorate, Egypt and acclimatized in large fiberglass tanks of 1500 L capacity containing aerated sea water (supplied from the same source as the fish) for 15 days before testing began. *T. zillii* was selected as a typical model of marine fish due to its simplicity of handling, whereas the handling of *M. seheli* is difficult. The water parameters were maintained within the permissible limits for *T. zillii*. The tank was filled with aerated sea water. Dissolved oxygen was monitored at 5 ± 1 mg L^−1^ using automatic air suppliers (RINA, Genova, Italy), while the water temperature was maintained at 27 ± 0.52 °C. The tank pH was regulated at 7.5, and a cycle of 13 h light/11 h dark was adopted. Ammonia and nitrite levels were measured twice a week and never exceeded 0.05 or 0.25 mg L^−1^, respectively. Moreover, six fish were randomly sampled and subjected to parasitological examination (gills and body surface were microscopically examined for the presence of parasites) and bacteriological examination. Only apparently healthy fish with no signs of disease were collected for experimental challenge.

#### 2.8.2. Experimental Setup

Approximately 120 acclimated *T. zillii* were assigned into 6 groups, each containing 2 subgroups (*n* = 10). Each subgroup contained 10 fish in 100 L capacity glass tanks. Five groups of fish (G1–G5) were experimentally challenged I/P with 0.2 mL sterile saline cotaining (3 × 10⁸ cfu/mL) virulent *A. veronii*. Each group was challenged with a corresponding *A. veronii* strain: Strain 1 harbored the *aer*A gene; Strain 2 harbored *aer*A and *ser* genes; Strain 3 harbored *aer*A, *ahp*, *ser*, and *nuc* genes; Strain 4 harbored *aer*A, *omp*AII, *alt*, *ahp*, and *act* genes; Strain 5ed harbored *aer*A, *alt*, *ahp*, *act*, *ser*, *nuc*, and *omp*AII genes. Another group (C: negative control) was I/P injected with sterile saline solution (0.85% NaCl). Five strains of *A. veronii* were selected and cultured on tryptic soy broth (Oxoid, UK) for inoculum preparation at 28 °C for 24 h. Then, the bacterial suspension was modified to the final concentration (3 × 10⁸ vcfu/mL) using a 0.5 McFarland standard as previously described [44]. The clinical signs, post-mortem findings, and mortality rates were checked for 14 days post-challenge as previously described [45]. To establish Koch’s postulates, dead fish were bacteriologically examined for bacterial re-isolation.

### 2.9. Statistical Analyses

The Chi-square test was applied to analyze the data frequencies using SAS software (version 9.4, SAS Institute, Cary, NC, USA); the level of significance was *p-*value< 0.05. Moreover, the correlations between antimicrobial drugs and antimicrobial resistance genes were determined using R-software (version 4.0.2; https://www.r-project.org/) (accessed on 1 July 2022).

## 3. Results

### 3.1. Clinical and Post-Mortem Findings

**Figure 1:**
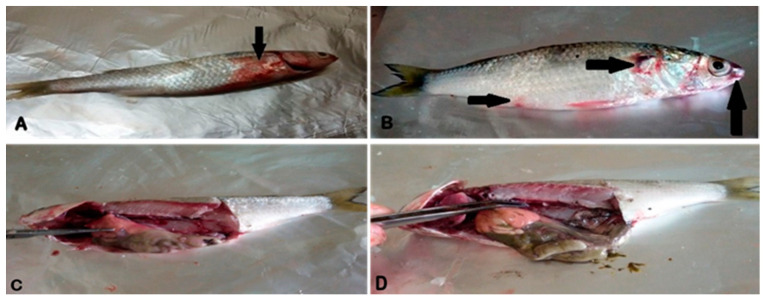
*M. seheli* showing (**A**): Erosion of gill cover and skin hemorrhages, (**B**): Erosion and hemorrhages in the opercular region, anus, and base of the fins, (**C**): Friable pale liver and congested kidney, (**D**): Congested kidney.

In the current study, the clinical inspection of naturally infected *M. seheli* revealed dark skin discoloration with detached scales (Figure 1A) and distinct hemorrhages at the base of the fins (Figure 1B). Moreover, the post-mortem findings of naturally infected *M. seheli* revealed hepatomegaly, friable liver with hemorrhagic patches (Figure 1C), and congested kidneys (Figure 1D).

### 3.2. Phenotypic Features and the Prevalence of A. veronii in the Examined M. seheli

All the recovered *A. veronii* isolates were Gram-negative, motile, straight rods. After 24 h at 28 °C, the bacteria grew effectively on the TSA medium, giving characteristically creamy, round, convex, shiny colonies. Colonies subsequently appeared green with black centers on Aeromonas-selective agar media. Moreover, the recovered colonies were convex, round, and hemolytic on blood agar, turning dark green after prolonged incubation. Biochemically, the obtained *A. veronii* isolates tested positive for oxidase, catalase, Voges–Proskauer, gelatin liquefaction, methyl red, casein, starch liquefaction, citrate utilization, and fermentation of glucose and sucrose. Moreover, the recovered *A. veronii* isolates were negative for H_2_S production, urea hydrolysis, bile esculin hydrolysis, nitrate reduction, and mannose fermentation.

**Figure 2:**
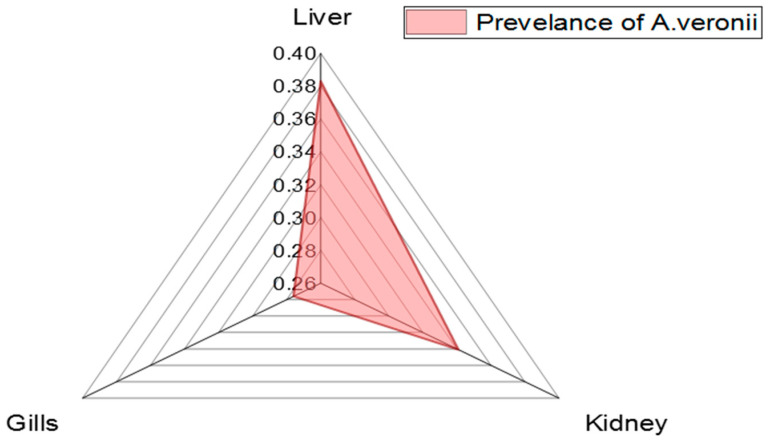
The distribution of *A. veronii* among different internal organs of naturally infected *M. seheli*.

The prevalence of *A. veronii* among the examined *M. seheli* was 22.5% (18/80). To measure the intensity of *A. veronii* among various examined organs of *M. seheli*, three different organs (liver, kidney, and gills) from the same fish were examined, with the highest prevalence noticed in the liver (38.3%), then the kidneys (34.1%), and gills (27.6%), as revealed in Table 2 and Figure 2. Statistically, there was no significant difference in the distribution of *A. veronii* among the examined internal organs of naturally infected *M. seheli* (*p*> 0.05).

### 3.3. 16srRNA Gene Sequencing and Phylogenetic Analyses

**Figure 3:**
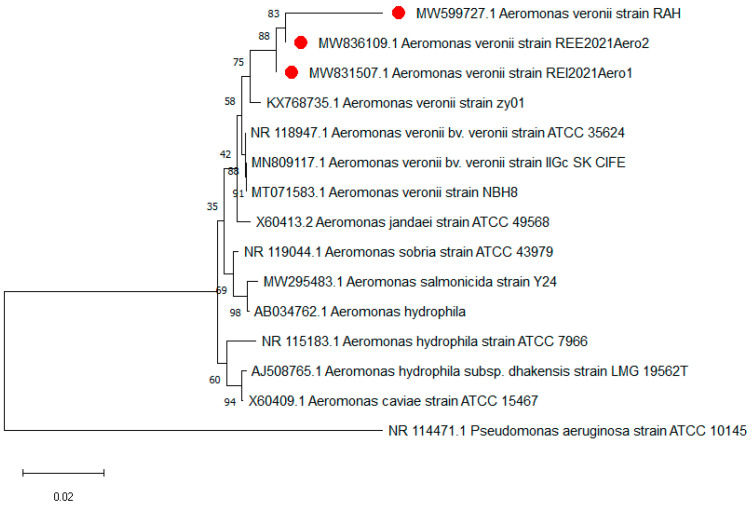
Phylogenetic tree based on *16S rRNA* gene sequencing. The tree clarifies the genetic relatedness of the tested *A. veronii* strains and other strains deposited in the GenBank database. The tested strains in the present study are indicated with red circles.

All the isolated *A. veronii* strains were positive for the *16srRNA* gene. The *16srRNA* gene sequencing showed that the tested *A. veronii* strains (accession nos.: MW831507, MW836109, and MW599727) had a common ancestor. Likewise, the tested *A. veronii* strains exhibited high similarity of genetic identity compared with other *A. veronii* strains from different sources, such as *A. veronii* strain zy01 (accession no.: KX768735) from China (94.5–98.9%), *A. veronii* strain ATCC35624 (accession no.: NR_118947) from UK (94.8–98.8%), *A. veronii* strain IIGc_SK_CIFE (accession no.: MN809117) isolated from Nile tilapia in India (94.8–98.8%), and *A. veronii* strain NBH8 (accession no.: MT071583) from China (94.8–98.8%), as illustrated in Figure 3.

### 3.4. Antimicrobial Resistance Profiles of the Retrieved A. veronii Isolates

**Figure 4:**
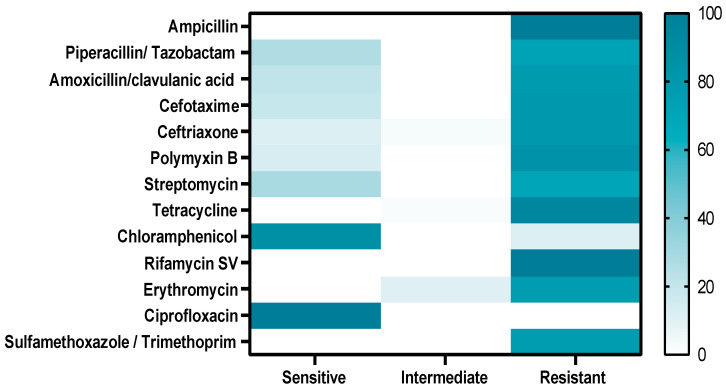
Antimicrobial susceptibility patterns of *A. veronii* isolates retrieved from examined *M. seheli* samples.

**Figure 5:**
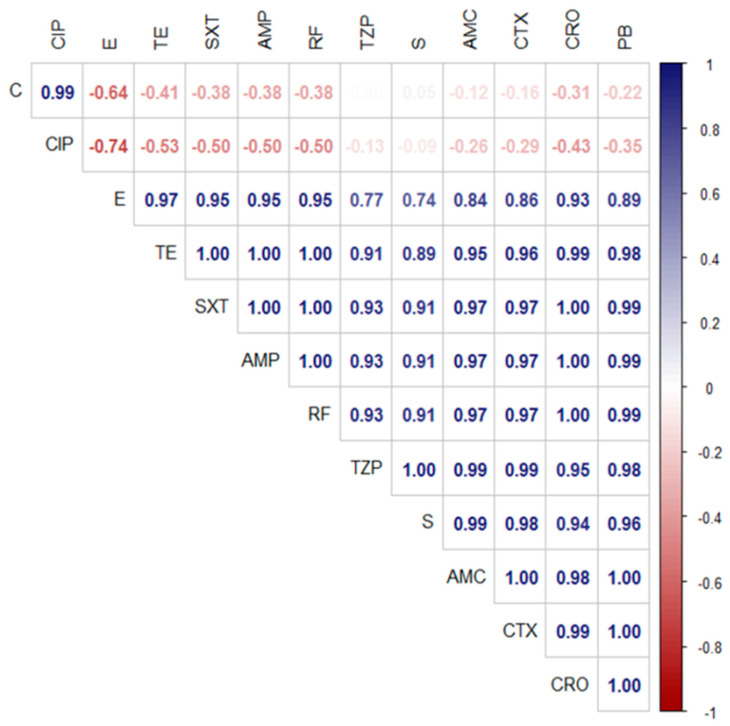
The correlation coefficient (r) between various tested antibiotics in the disc diffusion test.

The antimicrobial susceptibility testing revealed that the retrieved *A. veronii* strains showed significant resistance to different antimicrobial agents including ampicillin, rifamycin SV, sulfamethoxazole/trimethoprim (100% for each), tetracycline (95.7%), polymyxin B (85.1%), cefotaxime, ceftriaxone (80.9% for each), amoxicillin/clavulanic acid (78.8%), erythromycin (76.5%), piperacillin/ tazobactam (72.3%), and streptomycin (70.2 %). Moreover, ciprofloxacin (100%) and chloramphenicol (87.3%) revealed a potent antimicrobial activity against the retrieved *A. veronii* strains from *M. seheli*, as indicated in Table 3 and Figure 4. Significant differences (*p* < 0.05) were observed in the sensitivity of *A. veronii* isolates to various antibiotics, and remarkable positive correlations were recorded, e.g., TZP, S, and AMC (r = 0.99), SXT, RF, and PB (r = 0.99), TZP and CTX (r = 0.99), TE and CRO (r = 0.99), S and CTX (r = 0.98), TE and PB (r = 0.98), CRO and AMC (r = 0.98), SXT, AMP, RF, and AMC (r = 0.97), E and TE (r = 0.97), SXT, AMP, RF, and CTX (r = 0.97), PB and S (r = 0.96), TE and CTX (r = 0.96), and TE and AMC (r = 0.95), as illustrated in Figure 5.

### 3.5. Dissemination of Virulence-Determinant and Antimicrobial Resistance Genes in the Emerging A. veronii Strains

**Figure 6:**
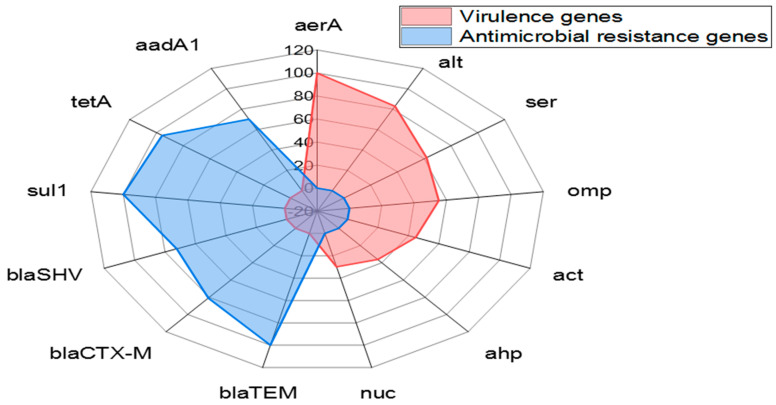
Distribution of virulence and antimicrobial resistance genes among *A. veronii* strains retrieved from the examined *M. seheli*.

The PCR indicated that the *aer*A gene (100%) was the principal virulence gene in *A. veronii* strains recovered from *M. seheli*, followed by *alt* (82.9%), *ser* (61.7%), *omp*AII (55.3%), *act* (44.7%), *ahp* (36.17 %), and *nuc* (29.8%) virulence genes. Likewise, the tested *A. veronii* strains carried the *bla*_TEM_, *sul*1, *tet*A, *bla*_CTX-M_, *bla*_SHV_, and *aad*A1 resistance genes with prevalence of 100%, 100%, 95.7%, 80.9%, 72.3%, and 70.2%, respectively, as revealed in Table 4 and Figure 6. A significant difference (*p* < 0.05) was noticed in the distribution of virulence-determinant genes in the obtained *A. veronii* strains. Conversely, there was no significant difference (*p* > 0.05) in the dissemination of resistance genes among the obtained *A.veronii*.

### 3.6. Genotypic and Phenotypic Multidrug-Resistance Patterns of the Emerging A. veronii Strains

**Figure 7:**
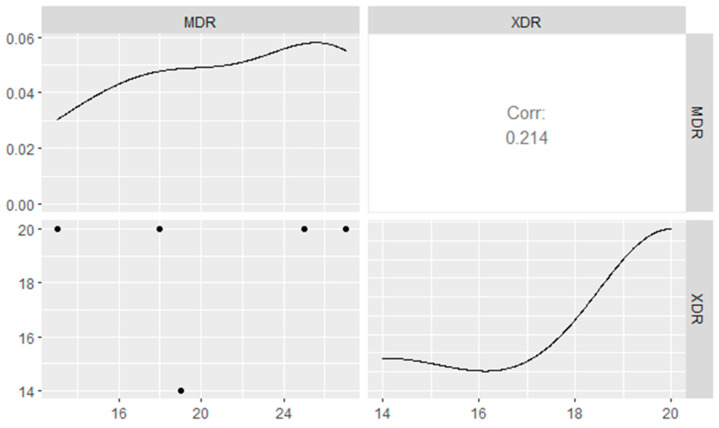
Distribution of XDR and MDR patterns among *A. veronni* strains isolated from the examined *M. seheli*. The horizontal axis indicates MDR and XDR patterns, while the vertical axis indicates the antimicrobial resistance genes.

**Figure 8:**
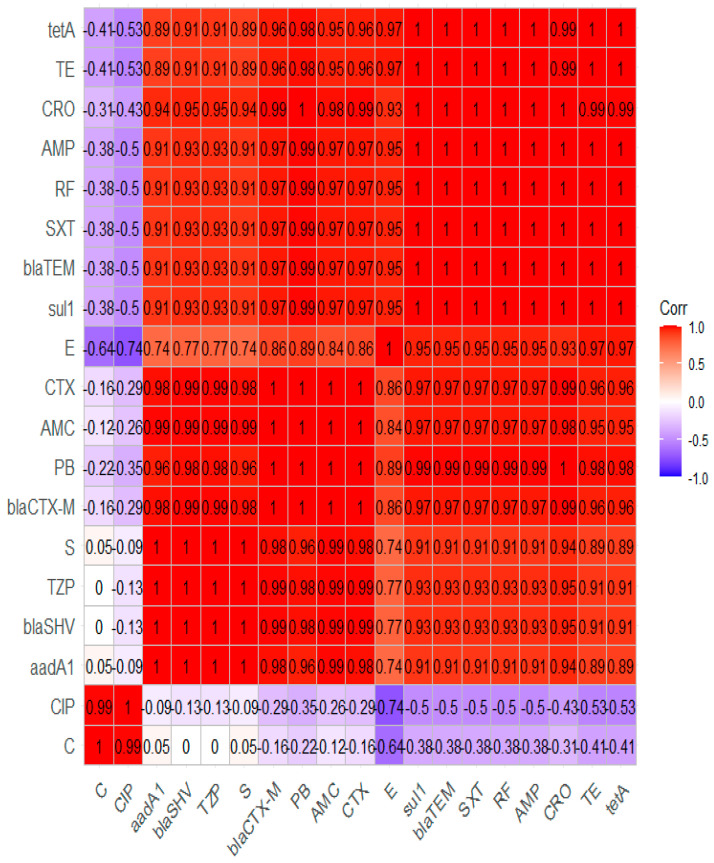
The correlation coefficient (r) between the tested antibiotics and the identified antimicrobial resistance genes.

Our findings revealed that 29.8% (14/47) of the recovered *A. veronii* strains were extensively drug-resistant (XDR) to nine classes and carried *bla*_TEM_, *bla*_CTX-M_, *bl*a_SHV,_ *tet*A, *aadA*1, and *sul*1 resistance genes. Likewise, 19.1% (9/47) of the obtained strains displayed multi-drug resistance (MDR) to eight antimicrobial classes and possessed *bla*_TEM_, *bla*_CTX-M_, *bla*_SHV,_ *tet*A, *aad*A1, and *su*l1 resistance genes. Meanwhile, 14.9% (7/47) of the recovered *A. veronii* strains were MDR to seven classes and carried *bla*_TEM_, *bl*a_SHV,_ *tet*A, and *sul*1 genes. In addition, 12.8% (6/47) of the obtained *A. veronii* strains were XDR to nine different classes and possessed *bla*_TEM_, *bla*_CTX-M,_ *aad*A1, *tet*A, and *sul*1 genes, as shown in Table 5 and Figure 7. Moreover, our findings revealed that the MAR index values were > 0.2, signifying that the *A. veronii* strains isolated from *M. seheli* originated from high-risk contamination. Furthermore, the correlation coefficient (r) between the resistance genes detected in *A. veronii* isolates and the tested antimicrobial agents was estimated. Remarkable positive correlations were recorded, including *bla*_TEM_ gene and AMP (r = 1), *bla*_CTX-M_ and CTX (r = 1), *tet*A gene and TE (r = 1), *sul*1 gene and STX (r = 1), *aad*A1 gene and S (r = 1), *bla*_SHV_ and TZP (r = 1), *bla*_CTX-M_ and CRO (r = 0.99), *bla*_SHV_ gene, AMC, and CTX (r = 0.99), *bla*_TEM_ gene and AMC (r = 0.97), *bla*_SHV_ gene and CRO (r = 0.95), *bla*_SHV_ and AMP (r = 0.93), and *bla*_TEM_ gene and TZP (r = 0.93), as shown in Figure 8.

### 3.7. Pathogenicity Test

**Figure 9:**
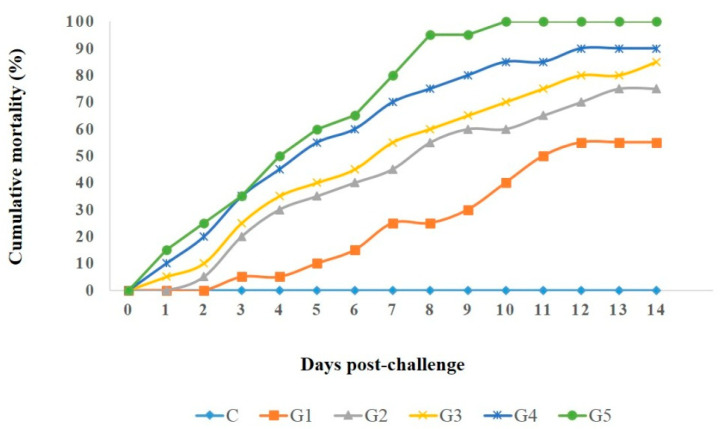
Cumulative mortality of *T. zillii* experimentally challenged with different virulent strains of *A. veronii* (G1-G5: Fish groups received a bacterial dose of 3 *×* 10⁸ cfu*/*mL of various *A. veronii* strains S1–S5, respectively), data were analyzed 14 days after challenge.

Five *A. veronii* strains (harboring one, two, four, five, and seven virulence genes, respectively) were selected for the pathogenicity test, as illustrated in Table 6. The clinical signs, pathological lesions, morbidity, and mortality rates in the different groups were monitored for 14 days after challenge. The results showed that fish in the control group had no deaths or pathologic lesions. In contrast, the other groups had substantial mortality rates and septicemic lesions, identical to those reported in naturally infected fish, including dark skin discoloration with detached scales, skin ulcers, and distinct hemorrhages at the base of the fins. Moreover, the mortality rate positively correlated with the virulence-determinant genes, and the highest mortality rate (100%) was recorded in the group (G5) inoculated with *A. veronii* Strain 5 which harbored seven virulence genes (as described in Figure 9). Clinically, the majority of infected fish showed detached scales, darkness of the skin, a hemorrhagic vent, slow movement, and hemorrhagic patches, mainly at the base of fins. Post-mortem inspection demonstrated that the tested fish exhibited characteristic septicemia, including enlarged kidneys, a congested liver, and accumulated bloody serous fluid in the abdominal cavity. Furthermore, *A. veronii* was re-isolated from various internal organs of the diseased and dead fish.

## 4. Discussion

*Aeromonads* are the most common septicemic bacterial fish pathogens, and are considered emerging food-borne pathogens associated with a significant threat to public health [46]. In the present work, the findings of clinical and post-mortem examinations were consistent with the results of [47,48,49], who observed congested gills, scattered hemorrhages on the skin, and detached scales, in addition to congested, friable and enlarged liver, and degenerative changes in the kidneys and spleen of fish naturally infected with *Aeromonads*. The degree of pathological alterations and the mortality rate are correlated with the severity of infection, fish immunity, and virulence determinants of *Aeromonas* species [50,51].

During the bacteriological examination, all retrieved isolates were recognized as *A. veronii* according to their morphological and biochemical features, and the recovered isolates revealed coordination of their phenotypic features. These results were consistent with those recorded by [6], who recovered *A. veronii* from *O. niloticus* in Egypt.

In the present study, *A. veronii* was recovered from moribund *M. seheli* with a prevalence of 22.5%, and the liver was the most predominant affected organ. The prevalence of *A. veronii* in this study was higher than that described by [6], who recorded only three isolates from diseased *O. niloticus*, and nearly similar to that reported by [11], who isolated 87 *A. veronii* strains from freshwater fish. *A. veronii* affects a variety of fish species and can live in environments where it may pose harm to the aquaculture industry and threaten food safety [52]. The prevalence of infection is attributed mainly to various predisposing variables, including stress resulting from fish density in intensive systems, poor management, poor hygienic conditions, poor water quality, insufficient oxygen, inappropriate pH, and temperature [14].

*Aeromonas* species are difficult to differentiate at the species level by conventional methods, due to the lack of a precise biochemical scheme to discriminate between them. Hence, molecular identification is essential for the differential diagnosis of *Aeromonas* species. The technique of *16S rRNA* sequencing is one of the most reliable molecular methods for identifying *A. veronii* [52]. In this study, all recovered isolates of *A. veronii* tested positive for *16S rRNA* using specific primers. Moreover, the *16SrRNA* phylogenetic analysis highlighted that the tested *A. veronii* strains originated from a common ancestor (accession nos: MW831507, MW836109, and MW599727). Furthermore, the tested *A. veronii* strains revealed a remarkable genetic similarity with other *A. veronii* strains from different geographical regions, such as *A. veronii* strain zy01 from China, *A. veronii* strain IIGc_SK_CIFE from India, *A. veronii* strain NBH8 from China, and *A. veronii* strain ATCC35624 from UK [53]. These results emphasize the epidemiological map and underline the public health significance of *A. veronii*.

Regarding the antibiogram profiling, ciprofloxacin showed an optimistic antimicrobial activity against the retrieved *A. veronii* strains from *M. seheli*. *Areomonads* are generally susceptible to fluoroquinolones [12]. In contrast, the retrieved *A. veronii* strains were highly resistant to sulfonamides, penicillin, tetracycline, cephalosporin, β-Lactam-β-lactamase-inhibitor combination, polymyxin, aminoglycosides, and macrolides. Our findings were similar to those recorded by [9,47]. The resistance of *A. veronii* to various antibiotics affects the health of animals and humans. Inappropriate application of antibiotics in the aquaculture system and the capability of *A. veronii* to obtain resistance genes from other MDR pathogens are the key predisposing causes contributing to the emergence of multiple drug-resistant superbugs. Therefore, regular use of antimicrobial sensitivity tests and screening for the existence of MDR strains are essential for selecting suitable antibiotics. The emergence of multidrug resistance in bacterial pathogens is attributed mainly to the propagation of antimicrobial resistance genes by horizontal transfer mediated by plasmids [54,55].

The detection of virulence-determinant genes is vital for understanding their potential pathogenicity and the prevention of probable infectious disease [56]. In this study, PCR revealed that the tested *A. veronii* strains frequently carried the *aer*A gene, followed by *alt*, *ser*, *omp*AII, *act*, *ahp*, and *nuc* virulence genes. Our findings are consistent with the results of [7,14,56]. Screening of virulence-determinant genes is a vital tool for identifying the possible pathogenicity of *Aeromonads* [57]. The pathogenicity of *A. veronii* is related to the expression of certain virulence determinants. Its pathogenicity is attributed mainly to the aerolysin toxin, cytotonic enterotoxins, serine proteases, outer membrane protein, and nuclease enzymes that are encoded by *aer*A, *alt*, *act*, *ser*, *ahp*, *omp*AII, and *nuc* genes, respectively [6,58]. The *aer* gene encodes for aerolysin toxin, which plays a significant role in the occurrence of infection. Aerolysin toxin is the primary virulence-determinant factor in *Aeromonads*, contributing to disease pathogenesis [7]. Moreover, cytotoxic enterotoxins (encoded by *alt* and *ac*t genes) and aerolysin toxin are essential virulence determinants for *Aeromonads*, and are categorized as potent foodborne pathogens. Both of these virulence determinants exert a substantial effect on the pathogenesis of disease [58]. Protease enzymes (encoded by *ser* and *ahp* genes) are common in *Areomonads*; they play a significant role in the proliferation of bacteria. Furthermore, they endorse the destruction of the mucosa and discoloration of the scales in fish, facilitating the invasion of bacterial pathogens. Serine proteases are characterized by potent caseinolytic activity [59]. The outer membrane proteins (encoded by the *omp*A gene) are responsible for mucosal adhesion in *A. veronii*. They exert a significant role in the attachment of *A. veronii* to the intestinal mucosa of the host [60].

Concerning the multi-drug resistance patterns in the retrieved *A. veronii* strains, a high percentage of the recovered *A. veronii* was XDR to nine different classes and carried *bla*_TEM_, *bla*_CTX-M_, *bla*_SHV,_ *tet*A, *aad*A1, and *sul*1 resistance genes. Furthermore, most of the isolated *A. veronii* were MDR to seven or eight different classes and possessed *bla*_TEM_, *bla*_CTX-M_, *bla*_SHV,_ *tet*A, *sul*1, and *aad*A1 resistance genes. Multi-drug resistance is thought to be one of the major hazards to public health across the world. It occurs due to the misuse of antibiotics in the aquaculture sector and in medical practice, and may include acquisition of antimicrobial resistance genes via mobile genetic elements [55,61,62,63]. The *bla*_TEM_ and *bla*_SHV_ resistance genes mainly mediate resistance to penicillin. Interestingly, the *bla*_TEM_ gene is the most predominant β-lactamase gene, commonly found in *Aeromonads* [14,64]. The resistance to sulfonamides and tetracycline is attributed mainly to the *sul*1and *tet*A resistance genes, respectively, which were the most predominant resistance genes found in this study. This was similar to the results of [64], who stated that tetracycline- and trimethoprim-resistance genes were demonstrated in all *A. veronii* genomes, an observation attributed mainly to the wide use of tetracycline and trimethoprim/sulfamethoxazole in the health sector and in veterinary settings. Moreover, the *bla*_CTX-M_ gene is responsible for cephalosporin resistance as well as resistance to β-Lactam-β-lactamase-inhibitor combinations. Furthermore, *aad*A1 is one of the most common aminoglycoside-resistance genes. The development of genes encoding antibiotic resistance on either the bacterial chromosome or plasmid is commonly attributed to the widespread unregulated use of antibiotics. The remarkable increase in antimicrobial resistance represents a rising obstacle in the treatment of diseases caused by MDR pathogens in humans and fish, and is considered a public health threat [40,55,65].

In the results of the pathogenicity tests, fish challenged with *A. veronii* showed different mortality rates that positively correlated with the prevalence of virulence genes in the inoculated strain. They exhibited typical clinical signs observed in naturally infected fish. These findings are similar to the results reported by [66]. The pathogenicity testing highlighted the virulence and pathogenicity of the *A. veronii* strains recovered from *M. seheli*. The pathogenicity tests revealed that the more virulence genes carried by a strain, the higher was the mortality rate.

## 5. Conclusions

In summary, to the best of our knowledge, this is the first study to have revealed the occurrence of XDR and MDR *A. veronii* strains in *M. seheli*. The recovered *A. veronii* strains commonly harbored the *aer*A, *alt*, *ser*, *omp*AII, and *act* virulence genes. The emerging *A. veronii* strains were XDR or MDR to several antimicrobial classes (for example, sulfonamides, penicillin, tetracycline, cephalosporin, β-Lactam-β-lactamase-inhibitor combination, polymyxin, aminoglycosides, and macrolides) and frequently carried *bla*_TEM_, *sul*1, *tet*A, *bla*_CTX-M_, *bla*_SHV_, and *aad*A1 resistance genes. Ciprofloxacin revealed optimistic antimicrobial activity against the XDR and MDR *A. veronii* strains retrieved from *M. seheli*. Conventional isolation methods and molecular assays are reliable epidemiological tools for identifying *A. veronii* in fish. Distressingly, the occurrence of XDR and MDR *A. veronii* strains is currently recognized as a public health threat, which moreover adversely affects the fish industry. Accordingly, regular practice of antimicrobial sensitivity tests and the proper use of antibiotics are called for in the aquaculture and health sectors.

## Figures and Tables

**Table 1:** List of primer sequences and PCR cycling conditions.

Gene	Primer Sequence	Cycling Conditions (35 Cycles)			Amplified Product	Reference
		Denatur.	Annealing	Extension		
*16SrRNA* *Aeromonas* species	F: CTACTTTTGCCGGCGAGCGG	94 °C for 30 s	50 °C for 40 s	72 °C for 50 s	953 bp	[30]
	R: TGATTCCCGAAGGCACTCCC					
*aer*A (Aerolysin)	F: CCTATGGCCTGAGCGAGAAG	94 °C for 30 s	55.5 °C for 30 s	72 °C for 30 s	431 bp	[36]
	R: CCAGTTCCAGTCCCACCACT					
*ser* (Serine protease)	F: ACGGAGTGCGTTCTTCCTACTCCAG	94 °C for 1 min	64 °C for 30 s	72 °C for 45 s	211 bp	[37]
	R: CCGTTCATCACACCGTTGTAGTCG					
*nuc* (Nuclease)	F: CAGGATCTGAACCGCCTCTATCAGG	94 °C for 1 min	64 °C for 30 s	72 °C for 45 s	504	
	R: GTCCCAAGCTTCGAACAGTTTACGC					
*act* Cytotoxic enterotoxin	F: GAGAAGGTGACCACCAAGAACA	94 °C for 4 min	59 °C for 30 s	72 °C for 1 min	232 bp	[18]
	R: AACTGACATCGGCCTTGAACTC					
*alt* (Heat-labile cytotonic enterotoxin)	F: TGACCCAGTCCTGGCACGGC	94 °C for 4 min	59 °C for 30 s	72 °C for 1 min	442 bp	[38]
	R: GGTGATCGATCACCACCAGC					
*ahp* (Serine protease)	F: ATTGGATCCCTGCCTA	94 °C for 4 min	59 °C for 30 s	72 °C for 1 min	911 bp	
	R: GCTAAGCTTGCATCCG					
*omp*AII (Outer membrane protein II)	F:GCTGAATTCATGAAACTCAAAATGGCTC	94 °C for 1 min	55 °C for 1 min	72 °C for 1 min	1001	[39]
	R: GCGAAGCTTTTACTGTTGTACTTGC					
*bla*_TEM_ (Penicillin-resistance)	F: ATCAGCAATAAACCAGC	94 °C 30 s	54 °C 40 s	72 °C 45 s	516	[40]
	R: CCCCGAAGAACGTTTTC					
*bla*_SHV_ (Penicillin-resistance)	F: AGGATTGACTGCCTTTTTG	94 °C 30 s	54 °C 40 s	72 °C 40 s	392	
	R: ATTTGCTGATTTCGCTCG					
*bla*_CTX-M_ (Cephalosporines-resistance)	F: ATGTGCAGYACCAGTAARGTKATGGC	94 °C 30 s	54 °C 40 s	72 °C 45 s	593	[41]
	R: TGGGTRAARTARGTSACCAGAAYC AGC GG					
*aad*A1 (Aminoglycosides-resistance)	F: TATCAGAGGTAGTTGGCGTCAT	94 °C 30 s	54 °C 40 s	72 °C 45 s	484	[42]
	R: GTTCCTAGCGTTAAGGTTTCATT					
*tet*A (Tetracycline-resistance)	F: GGTTCACTCGAACGACGTCA	94 °C 30 s	50 °C 40 s	72 °C 45 s	576	
	R: CTGTCCGACAAGTTGCATGA					
*sul*1 (sulfonamide-resistance)	F: CGGCGTGGGCTACCTGAACG	94 °C 30 s	54 °C 40 s	72 °C 45 s	433	[43]
	R: GCCGATCGCGTGAAGTTCCG					

**Table 2:** The intensity of *A. veronii* in different internal organs of naturally infected *M. seheli*.

Organ	No. of Positive Isolates	Percentage of Positive Isolates	Chi Square *p* Value
Liver	18	38.3%	0.80851 0.6675 ^NS^ *
Kidneys	16	34.1%	
Gills	13	27.6%	
Total	47	100%	

^NS^ * = Non-significant.

**Table 3:** Antimicrobial resistance profiles of the recovered *A. veronii* strains (*n* = 47).

Antimicrobial Class	Antimicrobial Agents	Sensitive		Intermediate		Resistant	
		*n*	%	*n*	%	*n*	%
**Penicillin**	Ampicillin	-	-	-	-	47	100
	Piperacillin/ Tazobactam	13	27.7	-	-	34	72.3
**β-Lactam-β-lactamase-inhibitor combination**	Amoxicillin/clavulanic acid	10	21.2	-	-	37	78.8
**Cephalosporin**	Cefotaxime	9	19.1	-	-	38	80.9
	Ceftriaxone	6	12.7	3	6.4	38	80.9
**Polymyxin**	Polymyxin B	7	14.9	-	-	40	85.1
**Aminoglycosides**	Streptomycin	14	29.8	-	-	33	70.2
**Tetracycline**	Tetracycline	-	-	2	4.3	45	95.7
**Phenicols**	Chloramphenicol	41	87.3	-	-	6	12.7
**Ansamycin**	Rifamycin SV	-	-	-	-	47	100
**Macrolides**	Erythromycin	-	-	11	23.4	36	76.5
**Fluroquinolones**	Ciprofloxacin	47	100	-	-	-	-
**Sulfonamides**	Sulfamethoxazole/Trimethoprim	-	-	-	-	47	100
	Chi square *p* value	252.82 <0.0001		92.875 <0.001		76.817 <0.0001	

**Table 4:** Prevalence of virulence and antimicrobial resistance genes in the retrieved *A. veronii* strains (*n* = 47).

Type of Genes	Genes	No of Positive Isolates	%	Chi-Square *p-*Value
**Virulence genes**	*aer*A	47	100	30.891 <0.0001
	*alt*	39	82.9	
	*ser*	29	61.7	
	*omp*AII	26	55.3	
	*act*	21	44.7	
	*ahp*	17	36.17	
	*nuc*	14	29.8	
**Antimicrobial resistance genes**	*bla*_TEM_	47	100	5.1475 0.3981 ^NS^ *
	*bla*_CTX-M_	38	80.9	
	*bla*_SHV_	34	72.3	
	*sul*1	47	100	
	*tet*A	45	95.7	
	*aad*A1	33	70.2	

^NS^ * = Non-significant.

**Table 5:** Distribution of phenotypic multi-drug resistance patterns and antimicrobial resistance genes among the retrieved *A. veronii* isolates.

No. of Isolates	%	Type of Resistance	Phenotypic Resistance	Resistance Genes	MAR
**14**	29.8%	XDR	Nine classes: AMP, TZP AMC RF SXT TE PB CRO, CTX S E	*bla*_TEM_,*bla*_CTX-M_, *bla*_SHV,_*tet*A, *aad*A1, and *sul*1	0.84
**9**	19.1%	MDR	Eight classes: AMP, TZP AMC RF SXT TE PB CRO, CTX S	*bla*_TEM_, *bla*_CTX-M_, *bla*_SHV_, *tet*A, *aad*A1, and *sul*1	0.76
**7**	14.9%	MDR	Seven classes: AMP, TZP AMC RF SXT TE PB E	*bla*_TEM_,*bla*_SHV_, *tet*A, and *sul*1	0.61
**6**	12.8%	XDR	Nine classes: AMP RF SXT TE PB CRO, CTX S E C	*bla*_TEM_, *bla*_CTX-M,_*aad*A1, *tet*A, and *sul*1	0.76
**5**	10.6%	MDR	Seven classes: AMP AMC RF SXT TE CRO, CTX E	*bla*_TEM_, *bla*_CTX-M_, *tet*A, and *sul*1	0.61
**4**	8.5%	MDR	Eight classes: AMP, TZP RF SXT TE PB CRO, CTX S E	*bla*_TEM_ *bla*_CTX-M,_*bla*_SHV,_*aad*A1, *tet*A, and *sul*1	0.76
**2**	4.2%	MDR	Four classes: AMP AMC RF SXT	*bla*_TEM_ and *sul*1	0.31

Piperacillin/tazobactam (TZP), ampicillin (AMP), amoxicillin/clavulanic acid (AMC), ceftriaxone (CRO), cefotaxime (CTX), polymyxin B (PB), tetracycline (TE), rifamycin SV (RF), erythromycin (E), chloramphenicol (C), streptomycin (S), ciprofloxacin (CIP), and sulfmethoxazole/trimethoprim (SXT).

**Table 6:** The cumulative mortality rate in different groups.

*A. veronii* Strains	Virulence Genes	Corresponding Group	Cumulative Mortality %
Strain 5	*aer*A, *alt*, *ahp*, *act*, *ser*, *nuc*, and *omp*AII	G5	100
Strain 4	*aer*A, *omp*AII, *alt*, *ahp*, and *act*	G4	90
Strain 3	*aer*A, *ahp*, *ser*, and *nuc*	G3	85
Strain 2	*aer*A and *ser*	G2	75
Strain 1	*aer*A	G1	55

## Data Availability

Not applicable.
